# The role of the dark triad and the experience of violence in the creation and dissemination of self-destructive online content by adolescents and youth

**DOI:** 10.1192/j.eurpsy.2022.2165

**Published:** 2022-09-01

**Authors:** G. Soldatova, S. Lyukhina

**Affiliations:** Lomonosov Moscow State University, Faculty Of Psychology, Moscow, Russian Federation

**Keywords:** self-destructive behavior, social media, self-injurious behavior, selfharm

## Abstract

**Introduction:**

Research in recent years has raised an important question about the role of the Internet in the self-injurious and suicidal behavior of adolescents and youth.

**Objectives:**

The aim of this work is to study the role of the experience of violence in real life and the dark triad in the creation and dissemination of self-destructive content among adolescents and youth.

**Methods:**

827 Russian students aged 15-25 (59% female) сompleted the questionnaire and the “Dirty Dozen” (Kornilova, et al., 2015).

**Results:**

Two-thirds of respondents have seen self-harm (72%) and suicidal (66%) content. Every seventh respondent (14%) is at risk, because he creates, approves with likes or disseminates self-destructive content among peers. Respondents at risk are more likely to have experienced physical (χ2=9.8, p<0.01), psychological (χ2=4.36, p<0.05) and sexualized (χ2=7.44, p<0.01) violence. Respondents who have a higher machiavellianism are more likely to approve (F=17.96, p=0.00) and disseminate (F=6.07, p<0.05) self-destructive content, less often using the «report» (F=4.06, p<0.05). Adolescents who have a higher psychopathic are more likely to create (F=7.34, p<0.01), disseminate (F=23.27, p=0.00) and approve (F=23.92, p=0.00) it.

**Conclusions:**

Self-destructive online content is seen by most teens and youth, and every seventh creates, approves and distributes it among peers, being a victim of violence in real life and having potential tendencies towards self-harm or suicidal behavior. Teens and youth with higher machiavellianism and psychopathy can create and disseminate self-destructive content due to their own psychological problems, but also potentially involve others. Research was supported by the Russian Foundation for Fundamental Research, project 20-013-00857.

**Disclosure:**

Research was supported by the Russian Foundation for Fundamental Research, project 20-013-00857.

